# Intra ventricular glioblastoma

**DOI:** 10.11604/pamj.2014.18.100.2788

**Published:** 2014-05-27

**Authors:** Cherkaoui Mandour, Brahim El Mostarchid

**Affiliations:** 1 Departement of neurosurgery, Military hospital Mohammed V, Rabat, Morocco

**Keywords:** Glioblastoma, brain, tumor

## Image in medicine

Glioblastoma represents 15%-20% of all intracranial tumors and approximately 50% of gliomas in adults. Although capable of arising anywhere in the central nervous system, these tumors mainly present as a frontotemporal lesion (63%) of the cerebral cortex. But, intraventricular glioblastoma is rare and only few cases have been reported in the literature. We report a case of 40-year-old woman who had a headache, vomiting and visual disturbances that persisted for four weeks. Magnetic resonance imaging showed an intraventricular lesion with inhomogeneous enhancement and infiltrative borders. These characteristics are consistent with other differential diagnoses: carcinomas, ependymomas and choroid plexus papillomas. The patient underwent a stereotactic biopsy allowed the final diagnosis of intra ventricular glioblastoma.

**Figure 1:**
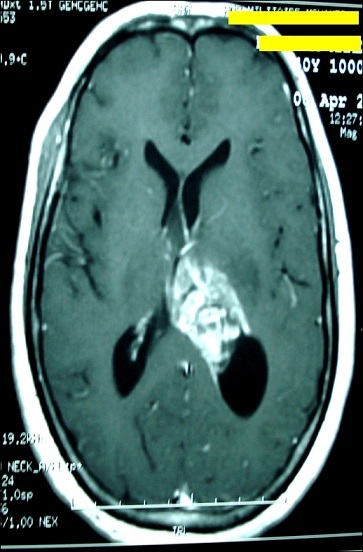
Magnetic resonance imaging showed an intraventricular lesion with inhomogeneous enhancement and infiltrative borders

